# Small Bowel Obstruction Caused by Carcinoid Tumor and Incidental Capsule Retention

**DOI:** 10.4021/gr240w

**Published:** 2010-11-20

**Authors:** Atul Singla, Todd Kilgore, Vanessa K. Kuwajima, Alberto Diaz-Arias, Matthew L. Bechtold

**Affiliations:** a Department of Internal Medicine, University of Missouri, Columbia, USA; b Department of Anatomical Sciences, University of Missouri, Columbia, USA

**Keywords:** Capsule endoscopy, Carcinoid tumor, Small bowel obstruction

## Abstract

Capsule endoscopy (CE) is a sensitive modality for examining the small bowel and is commonly employed to identify a variety of small bowel pathologies. We report a case of capsule retention leading to diagnosis of a chronic condition. A 60-year-old female presented with abdominal pain, nausea, and weight loss for 3 years. Physical exam revealed a mildly tender abdomen with hypoactive bowel sounds. Laboratory was normal. Abdominal radiographs showed a partial small bowel obstruction with retained capsule. Abdominal computed tomography (CT) demonstrated a retained capsule in the mid-portion of the jejunum, dilated small bowel, and terminal ileal mass. She underwent exploratory laparotomy showing an ileal mass with hepatic metastasis. A right hemicolectomy, reanastomosis, and removal of the retained capsule were performed. Pathology showed well-differentiated carcinoid tumor. She was discharged home for further treatment with oncology. Carcinoid tumors of the small bowel usually present with abdominal pain or small bowel obstruction. Our patient had intermittent small bowel obstruction due to a carcinoid tumor and retained capsule causing her symptoms. CE is a valuable tool but requires extensive consideration and possible patency capsule prior to use in patients presenting with symptoms consistent with Crohn’s disease or small bowel tumor.

## Introduction

Capsule endoscopy (CE) is a sensitive modality for examining the small bowel and is commonly employed to identify a variety of small bowel pathologies, from occult gastrointestinal bleeding to small bowel tumors [1-4]. Although CE has been shown to be relatively safe, a few contraindications are known. Relative contraindications to CE include difficulties swallowing capsule (dementia, esophageal stricture, Zenker’s diverticulum, and esophageal dysmotility), gastroparesis, partial or intermittent small bowel obstruction, presence of defibrillator or pacemaker, pregnancy, and patient’s refusing surgery or being poor operator candidates [5]. The capsule may be retained in up to 1.4% of studies [6]. Of these cases, patients with known Crohn’s disease experienced the highest incidence of capsule retention [6].

Although capsule retention is a known phenomenon, little is known about the effects of extensive long-term capsule retention [7]. Therefore, we report a unique case of extended capsule retention (around 3 years) which led to a diagnosis of a rare chronic condition.

## Case Report

A 60-year-old female with a three-year history of abdominal pain, nausea, and weight loss presented for evaluation of worsening symptoms. Over the previous three years, she had been evaluated with a contrast-enhanced computed tomography (CT) scan of the abdomen and colonoscopy, with intubation of the terminal ileum and random biopsies which failed to demonstrate abnormal pathologic findings. A concern for Crohn’s disease was raised and she subsequently underwent CE, revealing no abnormal findings. She was diagnosed with irritable bowel syndrome based on her chronic symptoms and lack of physical findings and was prescribed dicyclomine with minimal relief. After three years of her initial symptoms, she presented at our facility for a second opinion with worsening abdominal pain, nausea, and vomiting. She denied any constipation, diarrhea, hematochezia, rectal pain, facial flushing, or tenesmus. She reported no changes in her appetite but did have a 20-pound weight loss over the last three years.

Physical examination revealed normal vital signs including afebrile. Her jugular venous pressure and heart sounds were normal without S3, S4, rubs, or gallops. Abdomen was mildly tender with hypoactive bowel sounds without rebound tenderness or guarding. Neurologic examination revealed no focal deficits or nuchal rigidity. Laboratory evaluation revealed a white blood cell count of 4,900/µL (72% polymorphonuclear leukocytes), hemoglobin 12.5 g/dL, and platelets 186,000/µL. Complete metabolic panel revealed normal electrolytes, serum creatinine and liver function. Serum amylase and lipase were 40 U/L and 12 U/L, respectively. ESR was 12 mm/hr and TSH level was 1.15 mU/L.

**Figure 1:**
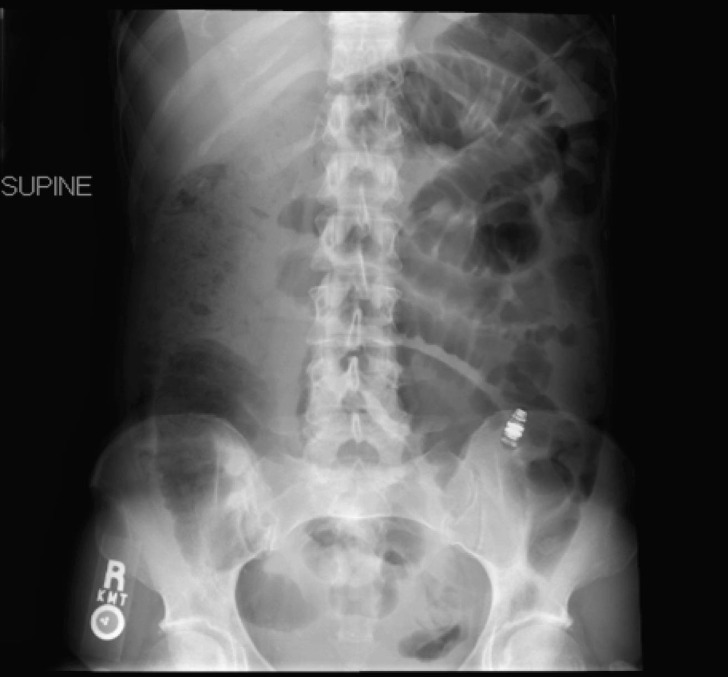
Abdominal radiograph showing a partial small bowel obstruction with retained capsule.

**Figure 2:**
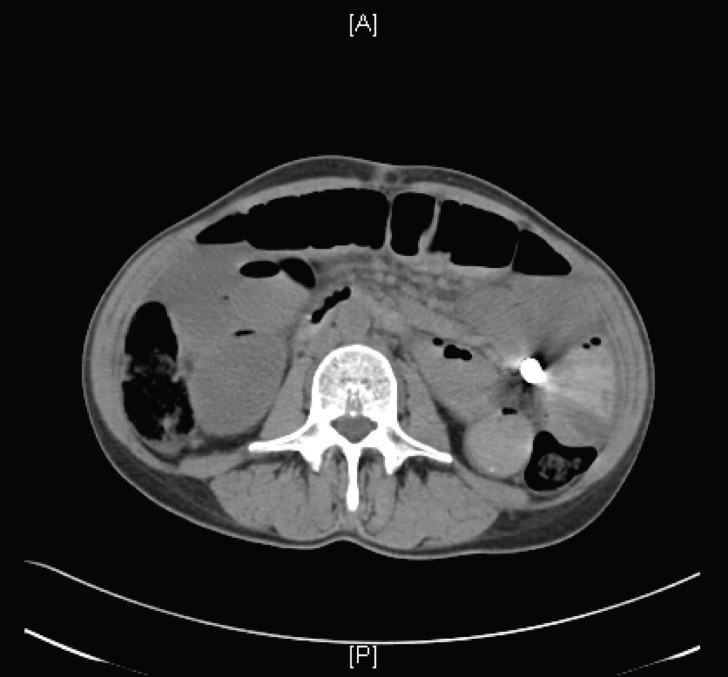
Abdominal CT demonstrating a retained capsule in the mid-portion of the jejunum.

On admission, abdominal radiographs showed a partial small bowel obstruction with retained capsule (Fig. 1). Abdominal CT scan demonstrated the retained capsule with a confirmed location in the mid-portion of the jejunum (Fig. 2). Dilated small bowel and terminal ileal mass were also noted; however, no cutoff point was observed at the retained capsule site. She was conservatively managed with continuous nasogastric suction, pain medications, and nothing per mouth. After 48 h of observation without any improvement, she underwent surgical exploration.

**Figure 3:**
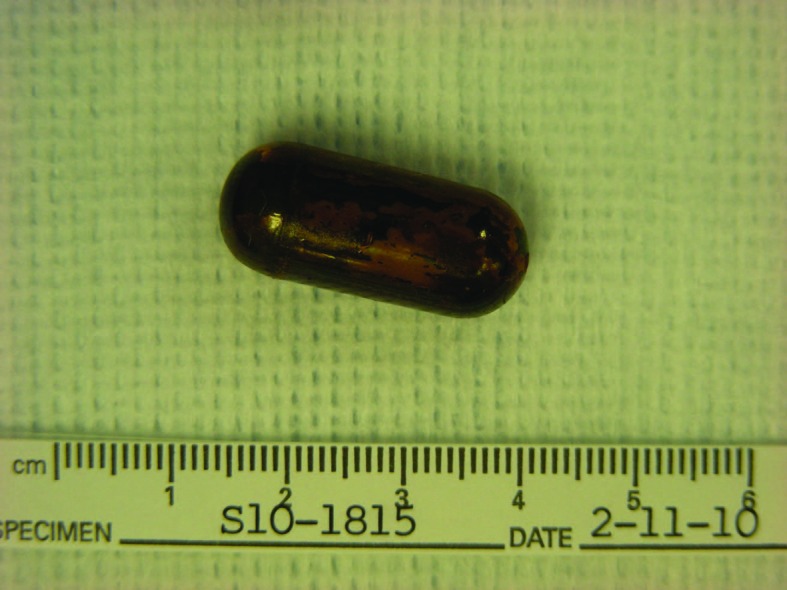
Photograph of retrieved capsule.

**Figure 4:**
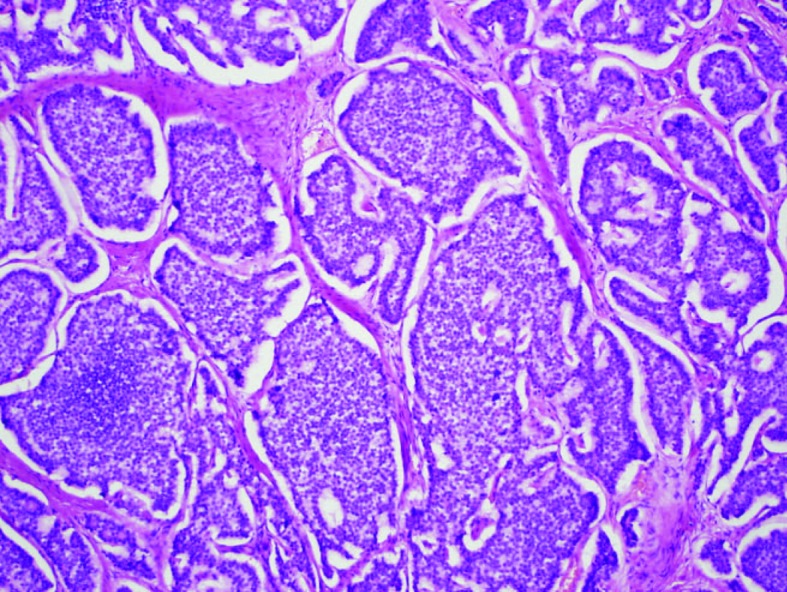
Carcinoid tumor with classic solid organoid and acinar patterns.

**Figure 5:**
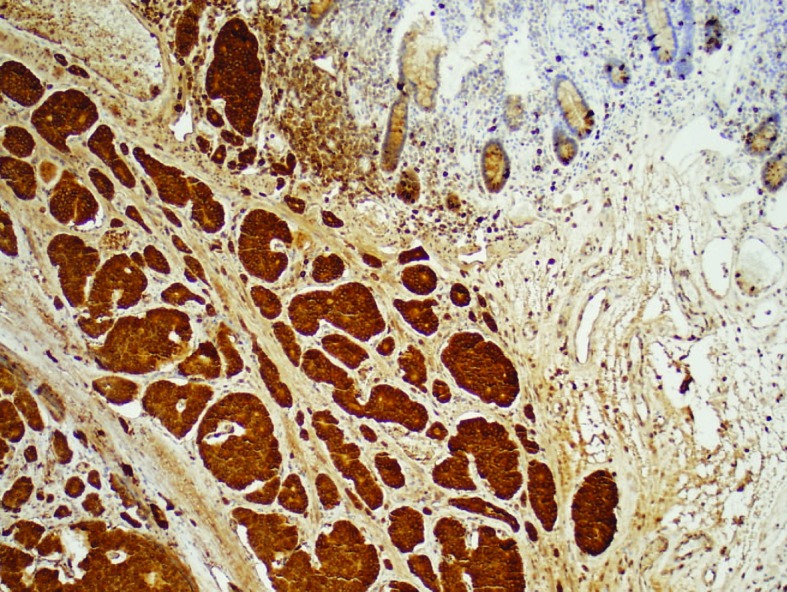
Carcinoid tumor with diffusely positive chromogranin.

Exploratory laparotomy revealed ileal tumor with metastasis to para-enteric lymph nodes and right lobe of the liver. She underwent right hemicolectomy, dissection of superior mesenteric artery, reanastomosis, and removal of the retained capsule (Fig. 3). Pathology showed well-differentiated carcinoid tumor (Fig. 4 and 5). Twenty-four hour urinary 5-hydroxyindoleacetic acid (5-HIAA) was 2 mg/L.

## Discussion

Carcinoid tumors of the small bowel represent approximately one-third of small intestine neoplasms and most commonly occur within 60 cm of ileocecal valve [8]. Patients usually present in their sixties or seventies with abdominal pain or small bowel obstruction. Bowel obstruction or abdominal pain occurs due to intussusception, the mechanical effect of the tumor, or mesenteric ischemia due to local fibrosis or angiopathy. About 5-7% of patients with small bowel carcinoids have carcinoid syndrome [8].

The symptoms of carcinoid syndrome include flushing, diarrhea, tachycardia or hypotension, bronchospasm, telangiectasia, and right-sided heart disease or failure [8]. Symptoms typically do not occur till the tumor has metastasized to lungs or liver [8]. The diagnosis of a carcinoid tumor often is coincidental with surgery performed for another reason. Nonetheless, there are useful diagnostic techniques that facilitate identification and localization of the tumor. For patients with vasoactive symptoms, measuring the urinary excretion of 5-HIAA and serum chromogranin A level is recommended [8]. For those with symptoms of bowel dysmotility, CT and magnetic resonance imaging (MRI) may be helpful [8].

Our patient had intermittent small bowel obstruction causing chronic abdominal pain secondary to the carcinoid tumor, retained capsule, or combination of the two. She had no history of facial flushing, sweating, bronchospasm, or diarrhea. She underwent CE for suspicion of Crohn’s disease and retained the capsule for approximately three years.

In general, wireless CE is a sensitive modality for examining the small bowel and is commonly employed to identify the source of small bowel hemorrhage, tumors, ulcerations, or inflammatory conditions. Although CE is usually safe, capsule retention due to an unsuspected obstructive lesion has been reported with overall incidence varying from 1% to 2% in multiple series [9]. Incidence of capsule retention in known Crohn’s disease is 13% versus 1.6% in suspected Crohn’s disease [10]. Nevertheless, CE made a definitive diagnosis in 12 of 31 cases (38.7%) of subacute small bowel obstruction where colonoscopy and gastroscopy could not identify the etiology [11]. In general, small bowel tumors are a significant finding at CE often missed by other investigations [12]. Capsule endoscopy is a valuable tool but requires extensive consideration and possible patency capsule prior to use in patients presenting with symptoms consistent with Crohn’s disease or small bowel tumor [13].

Our patient was discharged home after surgical resection with follow-up with oncology service. In general, small bowel carcinoid tumor with metastasis carries poor prognosis.

## References

[1] Pennazio M, Santucci R, Rondonotti E, Abbiati C, Beccari G, Rossini FP, De Franchis R (2004). Outcome of patients with obscure gastrointestinal bleeding after capsule endoscopy: report of 100 consecutive cases. Gastroenterology.

[2] Triester SL, Leighton JA, Leontiadis GI, Fleischer DE, Hara AK, Heigh RI, Shiff AD (2005). A meta-analysis of the yield of capsule endoscopy compared to other diagnostic modalities in patients with obscure gastrointestinal bleeding. Am J Gastroenterol.

[3] Costamagna G, Shah SK, Riccioni ME, Foschia F, Mutignani M, Perri V, Vecchioli A (2002). A prospective trial comparing small bowel radiographs and video capsule endoscopy for suspected small bowel disease. Gastroenterology.

[4] Liangpunsakul S, Chadalawada V, Rex DK, Maglinte D, Lappas J (2003). Wireless capsule endoscopy detects small bowel ulcers in patients with normal results from state of the art enteroclysis. Am J Gastroenterol.

[5] Storch I, Barkin JS (2006). Contraindications to capsule endoscopy: do any still exist?. Gastrointest Endosc Clin N Am.

[6] Liao Z, Gao R, Xu C, Li ZS (2010). Indications and detection, completion, and retention rates of small-bowel capsule endoscopy: a systematic review. Gastrointest Endosc.

[7] Kelley SR, Lohr JM (2009). Retained wireless video enteroscopy capsule: a case report and review of the literature. J Surg Educ.

[8] Pasieka JL (2009). Carcinoid tumors. Surg Clin North Am.

[9] Karagiannis S, Faiss S, Mavrogiannis C (2009). Capsule retention: a feared complication of wireless capsule endoscopy. Scand J Gastroenterol.

[10] Cheifetz AS, Kornbluth AA, Legnani P, Schmelkin I, Brown A, Lichtiger S, Lewis BS (2006). The risk of retention of the capsule endoscope in patients with known or suspected Crohn's disease. Am J Gastroenterol.

[11] Yang XY, Chen CX, Zhang BL, Yang LP, Su HJ, Teng LS, Li YM (2009). Diagnostic effect of capsule endoscopy in 31 cases of subacute small bowel obstruction. World J Gastroenterol.

[12] Bailey AA, Debinski HS, Appleyard MN, Remedios ML, Hooper JE, Walsh AJ, Selby WS (2006). Diagnosis and outcome of small bowel tumors found by capsule endoscopy: a three-center Australian experience. Am J Gastroenterol.

[13] Spada C, Shah SK, Riccioni ME, Spera G, Marchese M, Iacopini F, Familiari P (2007). Video capsule endoscopy in patients with known or suspected small bowel stricture previously tested with the dissolving patency capsule. J Clin Gastroenterol.

